# Resveratrol inhibits bile acid‐induced gastric intestinal metaplasia via the PI3K/AKT/p‐FoxO4 signalling pathway

**DOI:** 10.1002/ptr.6915

**Published:** 2020-10-25

**Authors:** Wenquan Lu, Zhen Ni, Shuqin Jiang, Mingfu Tong, Jian Zhang, Jing Zhao, Chenchen Feng, Qiaoyu Jia, Jingyun Wang, Tingting Yao, Hanbing Ning, Yongquan Shi

**Affiliations:** ^1^ Department of Gastroenterology The First Affiliated Hospital of Zhengzhou University Zhengzhou China; ^2^ State Key Laboratory of Cancer Biology, National Clinical Research Center for Digestive Diseases and Xijing Hospital of Digestive Diseases Air force Military Medical University Xi'an China; ^3^ Department of Gastroenterology The General Hospital of Western Theater Command Chengdu China; ^4^ Pediatric Development and Behavior Department The third Affiliated Hospital of Zhengzhou University Zhengzhou China; ^5^ Department of Gastroenterology Beijing Chao‐Yang Hospital, Capital Medical University Beijing China; ^6^ Department of Gastroenterology Second Affiliated Hospital of Xi'an Jiaotong University Xi'an China; ^7^ Postgraduate Department Xi'an Medical University Xi'an China

**Keywords:** bile acid, FoxO4, gastric intestinal metaplasia, PI3K/AKT, resveratrol

## Abstract

Gastric intestinal metaplasia (GIM) is the essential pre‐malignancy of gastric cancer. Chronic inflammation and bile acid reflux are major contributing factors. As an intestinal development transcription factor, caudal‐related homeobox 2 (CDX2) is key in GIM. Resveratrol has potential chemopreventive and anti‐tumour effects. The aim of the study is to probe the effect of resveratrol in bile acid‐induced GIM. We demonstrated that resveratrol could reduce CDX2 expression in a time‐ and dose‐dependent manner in gastric cell lines. A Cignal Finder 45‐Pathway Reporter Array and TranSignal Protein/DNA Array Kit verified that resveratrol could increase Forkhead box O4 (FoxO4) activity and that Chenodeoxycholic acid (CDCA) could reduce FoxO4 activity. Furthermore, bioinformatics analysis showed that FoxO4 could bind to the CDX2 promoter, and these conjectures were supported by chromatin‐immunoprecipitation (ChIP) assays. Resveratrol can activate FoxO4 and decrease CDX2 expression by increasing phospho‐FoxO4 nucleus trans‐location. Resveratrol could increase FoxO4 phosphorylation through the PI3K/AKT pathway. Ectopic FoxO4 expression can up‐regulate FoxO4 phosphorylation and suppress CDCA‐induced GIM marker expression. Finally, we found a reverse correlation between p‐FoxO4 and CDX2 in tissue arrays. This study validates that resveratrol could reduce bile acid‐induced GIM through the PI3K/AKT/p‐FoxO4 signalling pathway and has a potential reversing effect on GIM, especially that caused by bile acid reflux.

## Graphical abstract

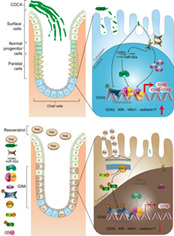

**Abbreviations**
AKT: also called PKB, protein kinase B
BCA: Bicinchoninic acid
CDCA: Chenodeoxycholic acid
CDX2: Caudal‐related homeobox 2
ChIP: Chromatin‐immunoprecipitation
DAB: Diaminobenzidine
DMSO: Dimethyl sulfoxide
FoxD1: Forkhead box D1
FoxO4: Forkhead box O4
FXR: Farnesoid X receptor
GC: Gastric cancer
GIM: Gastric intestinal metaplasia
HDCA6: Histone deacetylase 6
HNF4α: Hepatocyte nuclear factor 4 alpha
IHC: Immunohistochemistry
IIM: Incomplete intestinal metaplasia
IM: Intestinal metaplasia
Klf4: Kruppel‐like factor 4
Lipo 2000: Lipofectamine 2000
MAPK: Mitogen‐activated protein kinase
NF‐κB: Nuclear factor‐kappa B
PI3K: phosphoinositide 3‐kinase
Res: Resveratrol
RIPA: Radio immunoprecipitation assay
SHP: Small heterodimer partner

## 1 INTRODUCTION

Gastric cancer (GC) is the fifth highest incidence and third highest mortality in cancer‐related diseases among the world population (Bray et al., 2018; Etemadi, Safiri, Sepanlou, Ikuta, & Bisignano, 2020; Fitzmaurice et al., 2019), and it is the second most common type of cancer and the third leading cause of cancer‐associated mortality in China (Gao & Wu, 2019; Yang et al., 2018). Gastric intestinal metaplasia (GIM), especially incomplete type of intestinal metaplasia (IIM), characterized by caudal‐related homeobox 2 (CDX2) expression, is a significant premalignant lesion in the transition from chronic atrophic gastritis to GC (Nakayama et al., 2018; Rodrigues et al., 2018; Savcenko et al., 2019). In fact, many factors promote the development of GIM. Previous studies have shown that long‐term bile acid reflux and chronic inflammation are considered as important therapeutic factors (Hegyi, Maléth, Walters, Hofmann, & Keely, 2018; Li et al. 2019; Yu et al., 2019). The transition from IM to GC occurs over an average of 5–10 years (Correa, Haenszel, Cuello, & Ruiz, 1990; Correa, Haenszel, Cuello, Tannenbaum, & Archer, 1975). Therefore, IM stage can be an important broad time window to block the progression from gastritis to GC. Currently, there is no effective progress in the treatment or prevention of IM (Kim, 2019). Importantly, some natural compounds and small molecules that could be used for the treatment have been discovered.

GC incidence is lower in French than in China, because French people love to drink red wine almost every day among many factors (Bray et al., 2018; Etemadi et al., 2020). Studies have shown that a small amount of red wine can be beneficial for health. Resveratrol is a polyphenol compound in red wine. Previous studies have shown that resveratrol, usually extracted from grapes and vines, has a variety of antioxidant, anti‐inflammatory, and anti‐tumour effects (Chassot et al., 2018; Huminiecki & Horbańczuk, 2018; J. Xu et al., 2017; Y. Zhang, Cui, Wang, Gong, & Wang, 2019). Since it is easily accessible in nature, resveratrol is widely used in cosmetics and adjuvant drugs for tumours (Huminiecki & Horbańczuk, 2018; B. Xu et al., 2020; Zulueta, 2015). For example, there are many studies on resveratrol in liver cancer, colon cancer and GC (Farhadnejad, Emamat, & Zand, 2019; Fenner, 2017; Nana et al., 2018; Pistollato et al., 2017; Said, Mantawy, & El‐Demerdash, 2019). However, there is no research on resveratrol in IM. Therefore, this study investigates whether and how it affects IM and the underlying mechanism.

## 2 MATERIALS AND METHODS

### 2.1 Cell lines and cell culture

GES‐1, BGC823, SGC7901 and AGS cell lines were cultured in RPMI‐1640 (1X) (1640; C11875500BT, Thermo Scientific Gibco, USA) and were purchased from the American Type Culture Collection (ATCC, USA). MKN45, MKN28, AZ521 and HCT116 cell lines (obtained from ATCC, USA) were maintained in Dulbecco's modified Eagle's medium basic (1X) (DMED; C11995500BT, Thermo Scientific Gibco, USA). All media were supplemented with 10% foetal bovine serum (04‐001‐1A, Biological Industries, Israel). All cell lines were authenticated by STR DNA profiling and were tested for mycoplasma contamination. The cell lines were cultivated in a 37°C humidified incubator with 95% air and 5% CO_2_ (Thermo Scientific, China). Phosphate buffered saline, pH 7.4, basic (1X) (PBS; C10010500BT, Thermo Scientific Gibco, USA), was used as wash medium.

### 2.2 Cell transfection

HiPerFec transfection reagent (Lat No.160039992, Qiagen, USA) was used to transfect 100 nM of FoxO4 siRNA (Shanghai GenePharma Co., Ltd., China; the sequences for the FoxO4 siRNA are shown in Table 1) into GES‐1 and BGC823 cells for 24 hr according to the manufacturer's instructions. The cells were harvested for the next experiments at 24 hr post‐transfection. Furthermore, to obtain a stable cell line, AGS cells were infected with FoxO4 lentiviruses (multiplicity of infection, MOI = 40, Shanghai GeneChem Co. Ltd., China). Lipofectamine 2000 (Lipo 2000; Cat. No. 11668019, Thermo Invitrogen, USA) transfection reagent was used according to the manufacturer's instructions. The infection efficiency was detected by RT‐qPCR after selection with 2 μg/ml puromycin for 2 weeks. All transfection conditions used Opti‐MEM reduced serum medium (Opti‐MEM; No. 31985070, Thermo Scientific Gibco, USA) without antibiotics.

**TABLE 1:** FoxO4 siRNA sequences

Name	Sense(5′‐3′)	Antisense(5′‐3′)
FoxO4‐homo‐417	CGCGAUCAUAGACCUAGAUTT	AUCUAGGUCUAUGAUCGCGTT
FoxO4‐homo‐1,235	CAGCUUCAGUCAGCAGUUATT	UAACUGCUGACUGAAGCUGTT
FoxO4‐homo‐1820	GUGACAUGGAUAACAUCAUTT	AUGAUGUUAUCCAUGUCACTT
Negative control	UUCUCCGAACGUGUCACGUTT	ACGUGACACGUUCGGAGAATT

### 2.3 Reagents

Chemical reagents (obtained from Med‐Chem Express, MCE, USA) were dissolved in dimethyl sulfoxide (DMSO; Lot #SHBH2446V, Sigma‐Aldrich, USA) for storage in a −80°C freezer chenodeoxycholic acid (CDCA; Cat. No. HY‐76847), 200 μM; resveratrol (Res; Cat. No. HY‐16561), 200 μM; LY294002 (Cat. No. HY‐10108), 50 μM.

Anti‐β‐actin (A1978, 1:5000) was purchased from Sigma‐Aldrich (USA), and GAPDH (MB001, 1:5000) and tubulin β (BS1482M, 1:5000) were purchased from Bioworld Technology (USA). Primary antibodies against human CDX2 (ab76541, 1:1000), and FoxO4 (ab128908, 1:1000), FoxO4 (phospho‐Ser262, ab126594, 1:10000) were from Abcam (UK). Primary antibodies against human CDX2 (#12306, 1:1000), Klf4 (#12173, 1:1000), Villin‐1 (#2369, 1:1000), cadherin‐17 (#42919, 1:1000), FoxO4 (#9472, 1:1000), AKT (#9272, 1:1000), phospho‐AKT Ser473 (#9271, 1:1000) and phospho‐AKT Thr308 (#9275, 1:1000) were purchased from Cell Signalling Technology (USA).

### 2.4 Transcription factor array

The identities of the DNA pull‐down proteins were determined by an established procedure using a TranSignal Protein/DNA Array Kit (Panomics, USA) according to the manufacturer's protocol. In short, the pulled‐down proteins were incubated with TranSignal probe mix (a set of biotin‐labelled oligonucleotides corresponding to consensus sequences of 345 known transcription factors as shown in Tables S1 and S3) to allow the formation of DNA/protein complexes, which were then separated from the unbound probes by agarose gel electrophoresis. The probes in the complexes were extracted and hybridized to a TranSignal Array. The hybridized probes signals were visualized using the chemiluminescent imaging system provided with the TranSignal Protein/DNA Array Kit (Panomics, USA) and exposed to X‐ray film. For the CDCA treatment group, GES‐1 cells were incubated with 200 μM of CDCA for 24 hr. The negative control group was incubated with the same concentration of DMSO. Then, total RNA was extracted 24 hr later.

To use the Cignal Finder 45‐Pathway Arrays (CCA 901L; Qiagen, Germany; Tables S2 and S4) in the plate format, a reverse transfection method must be employed. This approach involved seeding the AGS cell line onto the transfection complexes (containing the Attractene transfection reagent [Qiagen, Germany] and test nucleic acids) on the first day following the manufacturer's protocol. Then, resveratrol was added to the cells the following day after 16–24 hr of transfection and incubated for 6 hr prior to assay development. The luciferase assay was carried out using the Dual‐Luciferase Reporter Assay System (Promega, USA) following the manufacturer's protocol for developing the assay.

### 2.5 Protein extraction and western blotting

Radio immunoprecipitation assay lysis buffer (RIPA; HY‐K1001, Med‐Chem Express, USA) containing protease inhibitor cocktail phosphatase inhibitor cocktail I and III, and deacetylase inhibitor cocktail (HY‐K0010, HY‐K0021, HY‐K0023, HY‐K0030, Med‐Chem Express, USA) was used to extract protein from the cell lines. Proteins were quantified using the bicinchoninic acid (BCA) method following the manufacturer's protocol (23227, Thermo Scientific, USA). The cell lysates were then mixed with SDS‐PAGE sample loading buffer (P0015, Beyotime, China) and stored in a ‐20°C freezer. Proteins were separated using a 7.5 to 12% TGX FastCast Acrylamide Kit (Lat No.1610171, 1610173, 1610175, Bio‐Rad Lab. Inc., USA). Then, the proteins were transferred onto a nitrocellulose membrane (T71623, Pall Corporation, Port Washington, USA) at 25 V for 7 min using Trans‐Blot Turbo Transfer Buffer (10026938, 690BR023295, Bio‐Rad Scientific, USA), and blocked for 2 hr with 10% non‐fat milk in 1× TBS/0.1% (v/v) Tween‐20 at room temperature. Primary antibodies were added and incubated at 4°C overnight. Each primary antibody was used according to the manufacturer's protocol. Grayscale analysis of the blot bands was performed using Image Lab software (version 5.1, USA), and the values (three for each band) were normalized to that of the β‐actin control.

### 2.6 Total RNA extraction and RT‐PCR

A Takara MiniBEST Universal RNA Extraction Kit (Takara, China) was used to extract total RNA from all cell lines according to the manufacturer's instructions. PCR primers for GAPDH, CDX2, Villin1, Klf4, cadherin17, FoxO4 and Muc2 were purchased from TsingKeBio (Shaanxi, China). The PCR primer details are shown in Table 2. Reverse transcription PCR was performed using a PrimeScript RT reagent kit (TaKaRa, China) according to the manufacturer's instructions. Quantitative real‐time PCR was performed using SYBR Premix Ex Taq II (TaKaRa, China). Fluorescence was detected with a CFX96 detection system (Bio‐Rad, USA). GAPDH levels were considered internal controls for the assays, and each sample was assayed in triplicate.

**TABLE 2:** PCR primers sequences

Gene	PCR primers
GAPDH	Forward: 5′‐ GCACCGTCAAGGCTGAGAAC ‐3′ Reverse: 5′‐ TGGTGAAGACGCCAGTGGA ‐3′
CDX2	Forward: 5′‐ TTCACTACAGTCGCTACATCACCA ‐3′ Reverse: 5′‐ CTGCGGTTCTGAAACCAGATT ‐3′
Klf4	Forward: 5′‐ GTGCCCCGAATAACAGCTCA ‐3′ Reverse: 5′‐ TTCTCACCTGTGTGGGTTCG ‐3′
Villin1	Forward: 5′‐ CGACTGCTACCTGCTGCTCTACAC ‐3′ Reverse: 5′‐ CGGCTTGATAAGCTGATGCTGTAA ‐3′
Cadherin17	Forward: 5′‐ AGCAGGTCACCAGACTGGGATAC ‐3′ Reverse: 5′‐ TCAGATGCTTGAGCACTTTCAACA ‐3′
MUC2	Forward: 5′‐ GATCATAGCACCTTGGATGGG ‐3′ Reverse: 5′‐ GGCACAGTCTGATGACCGG ‐3′
FoxO4	Forward: 5′‐ TGGGCTCAATCTCACCTCTTCC ‐3′ Reverse: 5′‐ AGAAGCACCCTTCTCCTGCTGA ‐3′

### 2.7 Tissue microarray and immunohistochemistry

GIM tissue microarrays were purchased from Alenabio Biotech (IC00011c; contains 49 cases of GIM arrays). Of these 49 IM tissues, 45 were male (91.8%). The median age of all 49 IM tissues was 46 years (range 32–78 years). Patients with mild intestinal metaplasia were 20 (40.8%), patients with moderate intestinal metaplasia were 14 (28.6%) and patients with severe intestinal metaplasia were 15 (30.6%). All 49 cases were HP negative when these specimens were taken. For the normal tissue samples, we obtained 12 biopsy specimens from normal patients who underwent gastroscopy biopsy at the First Hospital of Zhengzhou University (Zhengzhou, China). Of these 12 normal patients, 6 were male (50.0%). The median age of all 12 normal patients was 38 years (range 19–57 years). All patients were HP negative to eliminate the impact of HP infection. All specimens were verified by the Department of Pathology at the First Hospital of Zhengzhou University. All patients gave their informed consent prior to their inclusion in the study. All protocols in this research study were approved by the Medical Ethics Committee of Zhengzhou University.

For immunohistochemistry (IHC), target molecules were analysed on tissue microarray chips (Shanghai Outdo Biotechnology, China) using a CDX2 antibody (ab76541, 1:1000, Abcam, USA) and a phosphor‐FoxO4 Ser197 antibody (Cat. No. D155059, Sangon Biotech, China). The slides were then incubated with HRP‐conjugated secondary antibodies (Dako, Denmark). Next, the proteins were in situ with DAB chromogenic substrate (Dako, Denmark), followed by counter‐staining with haematoxylin. Two observers scored the IHC results in a blinded manner. The expression‐level score depended on the intensity and extent of the staining. Staining intensity was classified as follows: 0 for negative staining; 1 for weak staining; 2 for moderate staining; and 3 for strong staining. The percentage of stained cells per tissue was detected semi‐quantitatively as follows: 0 (<1%); 1 (1–25%); 2 (26–50%); 3 (51–75%); and 4 (>75%). The histological score (H‐score) was calculated for each sample by the following formula: H‐score = proportion score × intensity score.

### 2.8 Immunofluorescence

Cells were seeded on a four‐well Glass slide (PEZGS16, Millicell, Germany) for 4–6 hr, treated with or without chemicals, fixed with 4% paraformaldehyde for 30 min and permeabilized with 0.1% Triton X‐100 in PBS for 10 min. Blocking solution was applied for 30 min at room temperature. Then primary antibodies, anti‐CDX2 (ab76541, 1:500, Abcam, UK) and anti‐p‐FoxO4 Ser197 (Cat. No. D155059, 1:100, Sangon Biotech, China), were added for incubation at 4°C overnight. Alexa Fluor 488/Cy3‐conjugated secondary antibodies (EK012, EK021, 1:800) were added and incubated for 2 hr at room temperature. Immunostaining signals and DAPI‐stained nuclei (BD5010, 1:2000, Bioworld, USA) were scanned at room temperature using 3DHISTECH (Digital Pathology Company, Hungary). Blinded observers quantified the CDX2 and p‐FoxO4 S197 distribution on a per‐slide basis, in 100 cells of each condition. No imaging medium was used. For better visualization, the images were adjusted using the levels and brightness/contrast tools in Photoshop according to the guidelines for the presentation of digital data.

### 2.9 Dual‐luciferase reporter assays

We obtained 2000‐bp fragments of the CDX2 (NM_001265) promoter region from NCBI (https://www.ncbi.nlm.nih.gov/) and predicted them with the JASPAR2020 database (http://jaspar.genereg.net/; Table 3). Truncation experiments were used to verify the promoter region. Wild‐type (WT) and corresponding mutated FoxO4 promoter fragments, containing the predicted FoxO4 binding sites, were PCR‐amplified and cloned into the firefly luciferase reporter plasmid pGL3‐basic (Promega, USA). Luciferase activity was detected with a dual‐luciferase reporter assay kit (E1910, Promega, USA) 24 hr after transfection in GES‐1 cells treated with or without CDCA for 24 hr. Then, the ratio of firefly:Renilla luminescence was calculated for each well. Finally, the sample well ratio was normalized to the ratio of a control well, and the relative fold‐change of the CDCA group relative to the control group was calculated.

**TABLE 3:** Potential FoxO4 binding site in the CDX2 promoter

Name	Score	Relative score	Start	End	Strand	Predicted site sequence
FoxO4	12.7136	1.00000000278	1988	1994	+	gtaaaca
FoxO4	11.037	0.966854745832	1157	1163	+	ataaaca
FoxO4	11.037	0.966854745832	1671	1677	+	ataaaca
FoxO4	8.82526	0.923128392494	368	374	+	gaaaaca
FoxO4	8.5719	0.918119420008	1102	1108	+	ataaata

### 2.10 Chromatin immunoprecipitation

Chromatin‐immunoprecipitation (ChIP) assays were performed using an EZ ChIP Kit (Millipore, USA). Cells were cross‐linked with 1% paraformaldehyde for 10 min at 37°C, and the reaction was quenched with 2.5 M glycine for 5 min at room temperature. DNA was immunoprecipitated from the cell lysates with a FoxO4 antibody (Cat. #720154, Thermo Scientific Invitrogen, USA) and subjected to FoxO4 binding site amplification (Table 4). The amplified fragments were then analysed on an agarose gel. A non‐specific IgG antibody was used as a negative control.

**TABLE 4:** PCR primers sequences for ChIP

CDX2	PCR primers
ChIP control	Forward: 5′‐ TATTAACCCAGAAAGGTAGGC ‐3′ Reverse: 5′‐ TGGTGAAGACGCCAGTGGA ‐3′
ChIP 1	Forward: 5′‐ CTGGAAGTCCCCCAGGAGGAC ‐3′ Reverse: 5′‐ TCTGGGAGGTGTTTTTATCC ‐3′
ChIP 2	Forward: 5′‐ GAGAGTTTTAAAGATACAGGT −3′ Reverse: 5′‐ GTTTTAAACTACAGAAGTAATG ‐3′
ChIP 3	Forward: 5′‐ AGTAATTTCCTTGGGCAAGA ‐3′ Reverse: 5′‐ TGTTTATGCATTTGCAGGGAAG ‐3′

### 2.11 Statistical analysis

SPSS software (version 23.0, USA) was applied for subsequent statistical analyses. The data are shown as the mean ± *SEM*, and Student's *t* test was used for comparisons between two groups. The rank test was used for categorical variables, and the Shapiro–Wilk test was used to determine whether the continuous variables had a Gaussian distribution. The linear correlation coefficient (Pearson's) was used to evaluate the correlation between p‐FoxO4 and CDX2 protein expression levels in IHC.

## 3 RESULTS

### 3.1 Resveratrol inhibits the expression of CDX2 and downstream intestinal markers in a time‐ and dose‐dependent manner in GC cells

**FIGURE 1:**
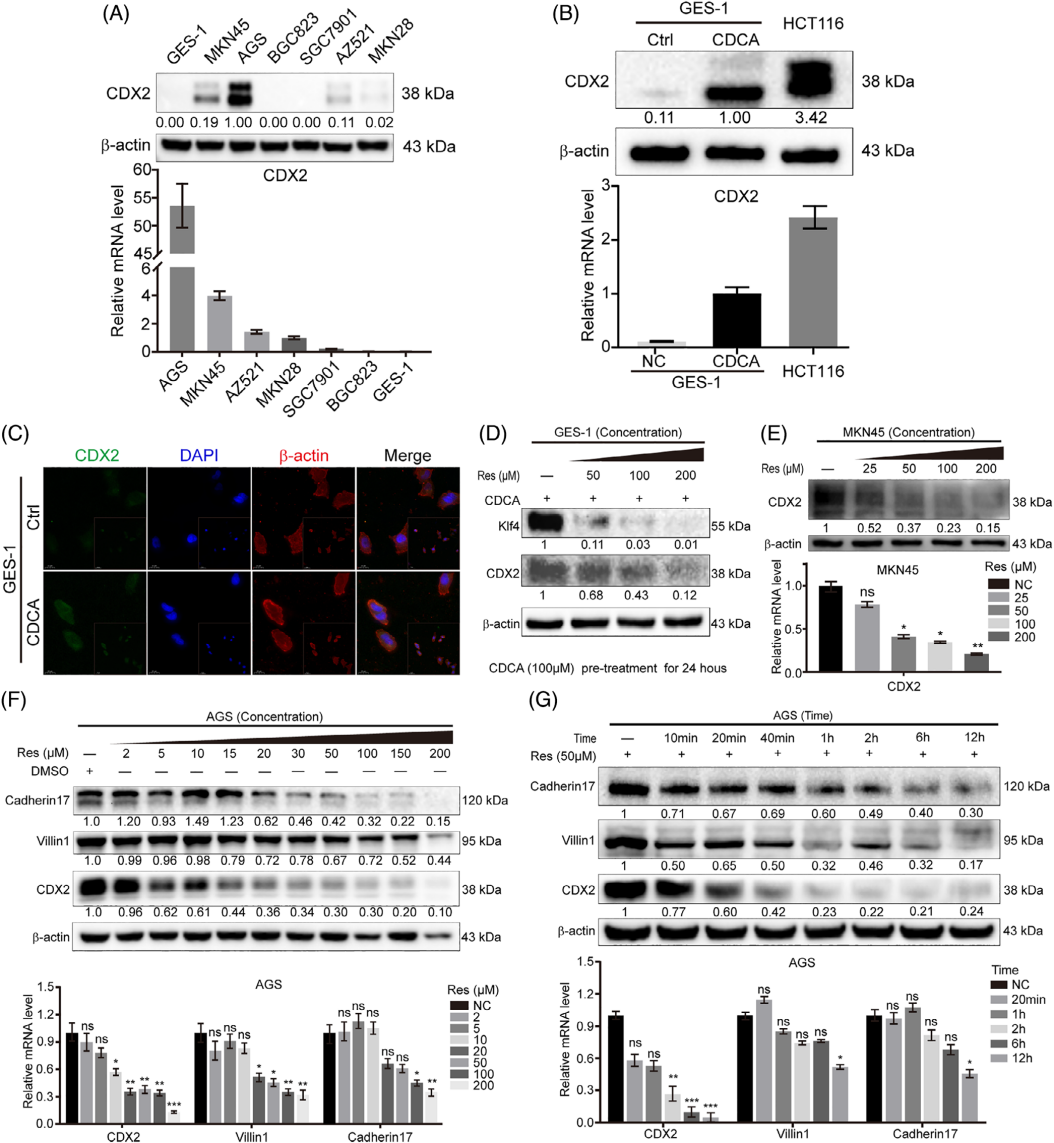
Resveratrol inhibits the expression of CDX2 and downstream intestinal markers in gastric cells and bile acid‐induced GIM. (A) The basic expression of CDX2 was measured at the protein (upper) and mRNA levels (lower) in GC cells and gastric epithelial cells (GES‐1). (B) The protein (upper) and mRNA (lower) expression levels of CDX2 were measured after CDCA stimulation (incubation time: 24 hr; dosage: 200 μM). The intestinal cancer cell line, HCT116, served as a positive control. (C) Cell immunofluorescence showed CDX2 expression in CDCA‐induced GIM (incubation time: 6 hr; dosage: 200 μM). Green: CDX2, Blue: Nuclei, red: Cytoskeletal protein. (D) Resveratrol down‐regulated CDX2 and Klf4 protein expression in a dose‐dependent manner in CDCA‐induced GIM (dosage of CDCA: 50 μM, Pre‐treatment for 24 hr) according to western blot analyses. (E–G) Resveratrol decreased the expression of CDX2 and downstream intestinal markers in a time‐ and dose‐dependent manner in AGS and MKN45 cells at the protein (upper) and mRNA (lower) levels. Unless otherwise specified, β‐actin, tubulin‐β and GAPDH were used as default western blot and RT‐PCR internal references. *p < .05; **p < .01; ***p < .001; ns, not significant [Colour figure can be viewed at wileyonlinelibrary.com]

The basic expression of CDX2 was measured at the protein and mRNA levels in gastric cells (Figure 1A). AGS and MKN45 cell lines have high expression of CDX2 molecules, while other cell lines have low expression. CDX2 expression was low in GES‐1 cells and high in AGS and MKN45 cells. Our previous work showed that CDX2 expression was dramatically increased after CDCA stimulation at the protein and mRNA levels (Figure 1B); in fact, they were close to the levels in the positive control HCT116 intestinal cells. Furthermore, the immunofluorescence results illustrated that CDCA induced CDX2 expression in the cytoplasm and nucleus of GES‐1 cells (Figure 1C). These results suggest that CDCA could increase CDX2 in GES‐1 cells. Surprisingly, resveratrol was able to inhibit the CDCA‐induced increase in CDX2 and the downstream intestinal marker KlF4 (Figure 1D) at the protein level. In GC cells, resveratrol reduced CDX2 expression (Figure 1E) at the protein and mRNA levels. Similarly, resveratrol downregulated CDX2 and downstream intestine‐specific markers in a time‐ and dosage‐dependent manner at the protein and mRNA levels (Figure 1F,G). Collectively, these results suggest that resveratrol may be a good candidate for treating or reversing IM.

### 3.2 FoxO4 was identified by the Cignal Finder 45‐pathway reporter array and TranSignal protein/DNA array kit and may combine directly with CDX2 promoter, as predicted by bioinformatics analysis

**FIGURE 2:**
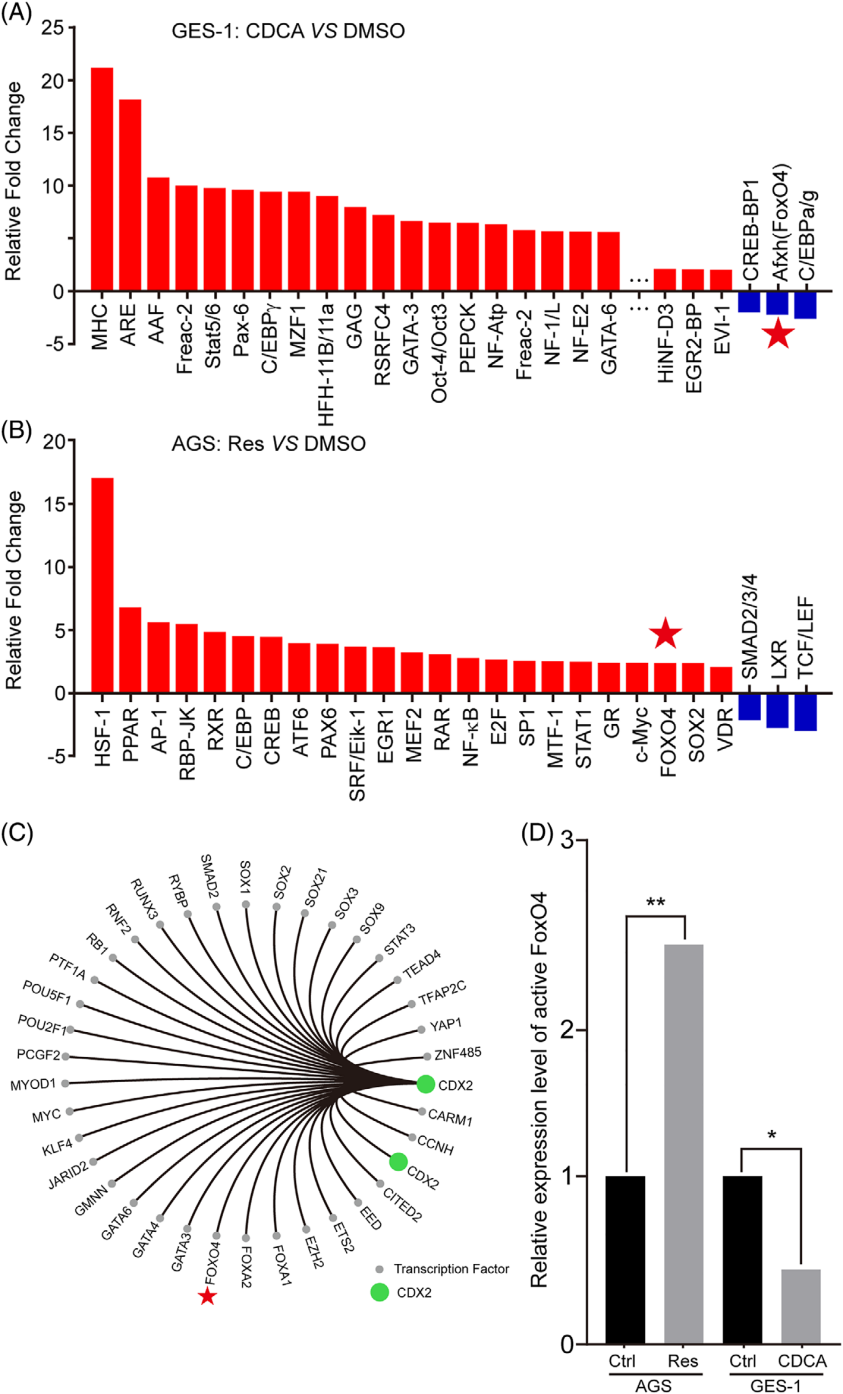
FoxO4 was identified by the Cignal Finder 45‐Pathway Reporter Array and TranSignal Protein/DNA Array Kit and may combine directly with the promoter region of CDX2 as predicted by bioinformatics analysis. (A) After CDCA treatment of GES‐1 cells, changes in the activity of various transcription factors were determined using the TranSignal Protein/DNA Array Kit (fold regulation >2.0 and *p* value <.05). Red: increased fold regulation, blue: decreased fold regulation. Incubation time: 24 hr; dosage: 200 μM. For more detailed data, please see Tables S1 and S3. (B) After resveratrol treatment of AGS cells, changes in the activity of various transcription factors were determined using the Cignal Finder 45‐Pathway Reporter Array (fold regulation >2.0 and *p* value <.05). Red: increased fold regulation, blue: decreased fold regulation. Incubation time: 24 hr; dosage: 200 μM. For more detailed data, please see Tables S2 and S4. (C) Transcription factors predicted by bioinformatics that could bind directly to the CDX2 promoter region. Green: CDX2, grey: transcription factors other than CDX2 with predicted direct binding to the CDX2 promoter. Red pentagram: FoxO4 or active FoxO4. (D) Relative activity change of the transcription factor FoxO4 after treatment with resveratrol and CDCA in gastric cells [Colour figure can be viewed at wileyonlinelibrary.com]

To further identify the functional targets involved in resveratrol‐induced CDX2 down‐regulation in CDCA‐stimulated GES‐1 and AGS cells, a pathway reporter array and protein/DNA array kit were performed. We found that resveratrol could increase FoxO4 activity upon CDCA stimulation, thus reducing the activity of transcription factors (Figure 2A,B, Tables S1–S4). The red pentagram indicates active FoxO4. Moreover, we used a bioinformatics analysis to predict transcription factors that can bind directly to the CDX2 promoter, and found FoxO4 can adapt to this result (Figure 2C, Table S5). Then, we determined the changes in active FoxO4 (Figure 2D). Together, these results suggest that active FoxO4 may play an essential role in the effects of resveratrol on reducing CDX2 expression.

### 3.3 Resveratrol activated FoxO4 through phosphorylation and nuclear trans‐location

**FIGURE 3:**
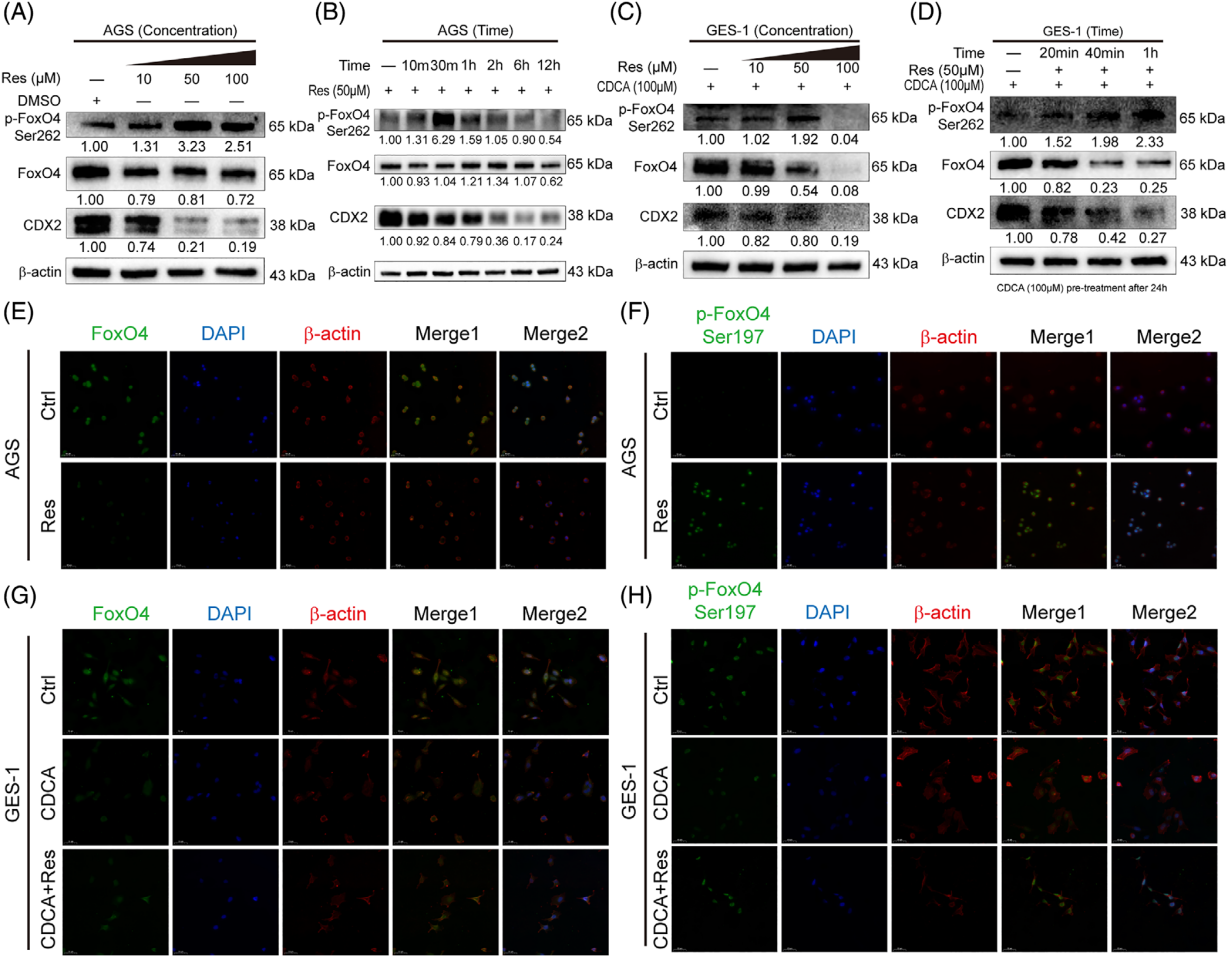
Resveratrol activated FoxO4 through phosphorylation and nuclear trans‐location. (A–D) The effect of resveratrol on FoxO4 activation was time‐ and concentration‐dependent at the protein level in AGS cells (A,B) and CDCA‐induced GIM (C, D). (E,F) Cell immunofluorescence showing the expression of FoxO4 and p‐FoxO4 Ser197 in the AGS cell line treated with or without resveratrol (incubation time: 6 hr; resveratrol dosage: 200 μM). Green: FoxO4 or p‐FoxO4 Ser197, blue: nuclei, red: cytoskeletal protein. (G,H) Cell immunofluorescence showing the expression of FoxO4 and p‐FoxO4 Ser197 in CDCA‐induced GIM with or without resveratrol treatment (incubation time: 6 hr; resveratrol dosage: 200 μM). Green: FoxO4 or p‐FoxO4 Ser197, blue: nuclei, red: cytoskeletal protein [Colour figure can be viewed at wileyonlinelibrary.com]

Next, we aimed to further investigate the mechanism of FoxO4 activation after resveratrol stimulation. We found that p‐FoxO4 Ser262 was increased in a time‐ and dosage‐dependent manner. However, there was no obvious change in regular FoxO4 at the protein level in the AGS cell line (Figure 3A,B). Similar to this result, the same pattern of p‐FoxO4 Ser262 expression was found in CDCA‐induced GES‐1 cells (Figure 3C,D). Moreover, FoxO4 expression was significantly reduced. Cell immunofluorescence results revealed that resveratrol increased FoxO4 Ser197 phosphorylation and reduced the increase in CDX2 induced by CDCA, but the FoxO4 changes were not consistent (Figure 3E–H). Collectively, these results illustrate that FoxO4 works by increasing its phosphorylation and nuclear trans‐location.

### 3.4 FoxO4 inhibits CDX2 expression by directly targeting the CDX2 promoter region which was screened by bioinformatics prediction and ChIP

**FIGURE 4:**
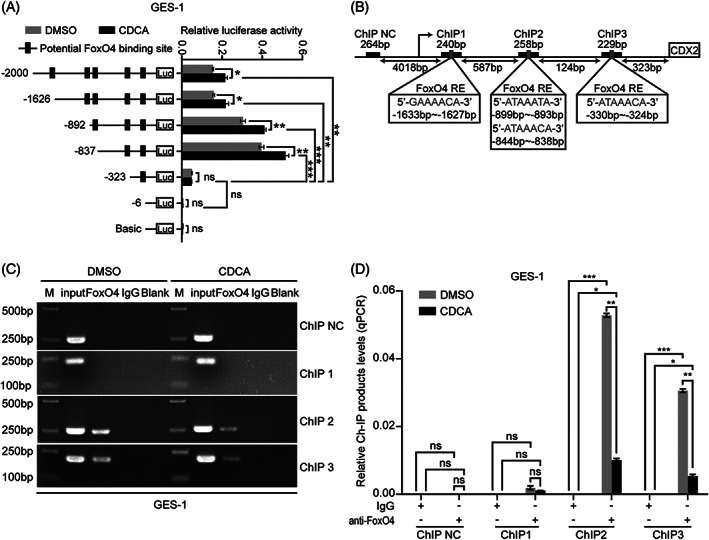
FoxO4 inhibits CDX2 expression by directly targeting the CDX2 promoter region. (A) Serially truncated CDX2 promoter constructs were cloned into pGL3‐luciferase reporter plasmids and transfected into GES‐1 cells. Four hours after transfection, the cells were treated with CDCA (200 μM) for 24 hr, and the relative luciferase activities were determined 72 hr later. (B) A ChIP assay demonstrated the direct binding of FoxO4 to the CDX2 promoter in GES‐1 cells. M: Marker. (C,D) qRT‐PCR of the ChIP products validated the binding capacity of FoxO4 to the CDX2 promoter. Means ± *SEM* of a representative experiment (*n* = 3) performed in triplicate is shown. *p < .05; **p < .01; ***p < .001; ns, not significant

We further elucidated the connection between FoxO4 and the CDX2 signalling pathway. Reporter genes, containing the CDX2 promoter, were transfected into GES‐1 cells, which were then treated with CDCA. This analysis revealed that CDCA‐based CDX2 regulation was controlled by potential FoxO4 binding sites located between −2000 and −323 bp (Figure 4A). ChIP assays further confirmed that FoxO4 binds to three regions (ChIP 2: −899 to −893 bp and −844 to −838 bp; ChIP 3: −330 to −324 bp) (Figure 4B). After the CDCA addition, CDX2 promoter activity was up‐regulated because of the reduction in FoxO4 binding to the CDX2 promoter region (Figure 4C,D). These results revealed that FoxO4 regulates CDX2 expression by binding to its promoter in gastric cells.

### 3.5 CDX2 expression was negatively regulated by p‐FoxO4

**FIGURE 5:**
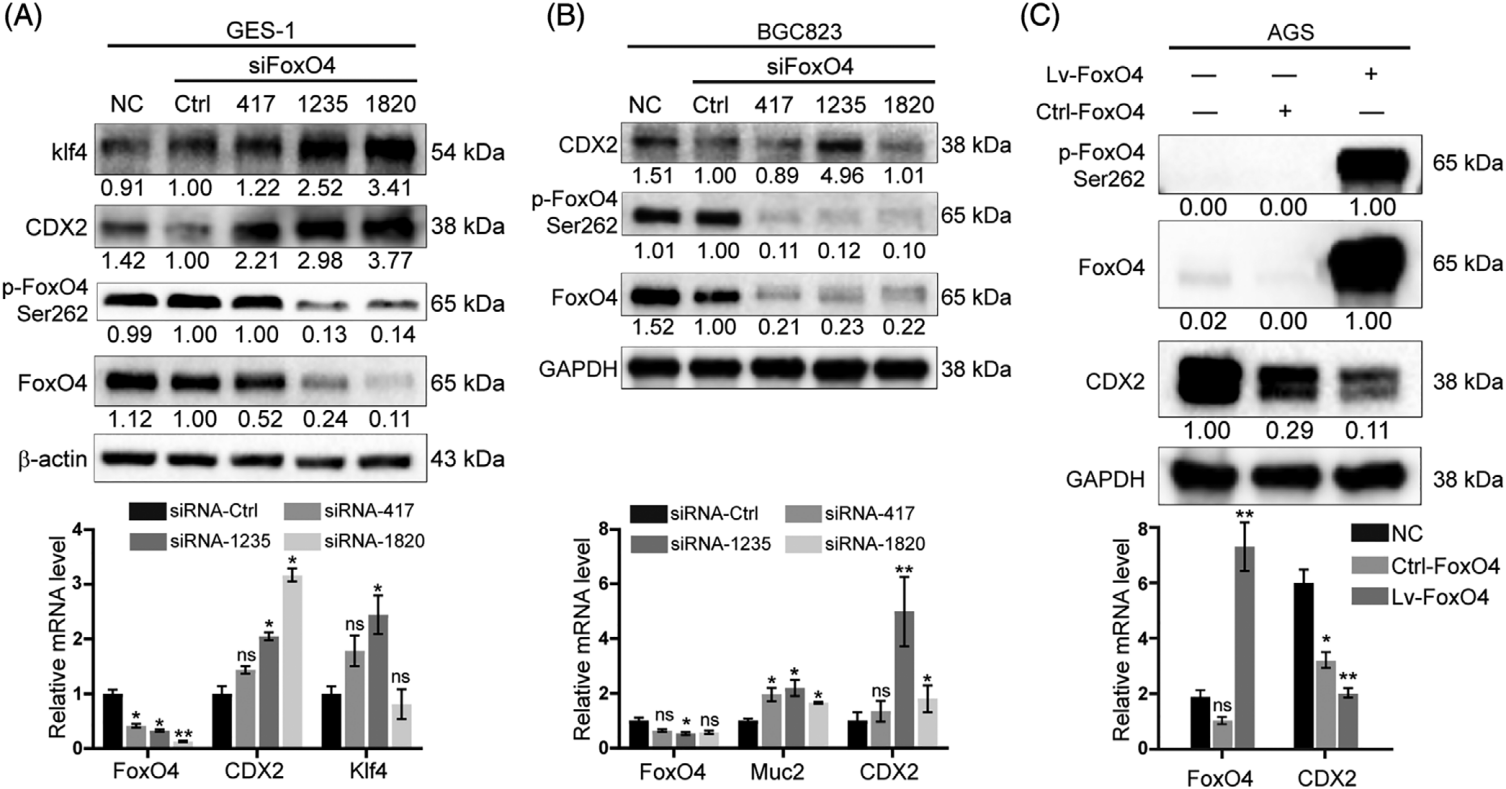
CDX2 expression was negatively regulated by p‐FoxO4. (A‐B) GES‐1 and BGC823 cells were transfected with FoxO4 small interfering RNA (siFoxO4). With the decrease in p‐FoxO4 Ser262, the expression of CDX2 and intestinal markers increased at the protein (upper) and mRNA (lower) levels. (C) AGS cells were transfected with FoxO4 lentivirus. The absolute increase in p‐FoxO4 Ser262 could reduce the expression of CDX2 at the protein (upper) and mRNA (lower) levels

To identify the relationship between p‐FoxO4 and CDX2, GES‐1 and BGC823 cells, which have low internal CDX2 expression, were chosen for siFoxO4 transfection to observe the changes in intestine‐specific markers. We found that upon the absolute decrease in endogenous p‐FoxO4, CDX2 and intestinal markers no longer had inhibitory effects and significantly increased at the protein and mRNA levels in GES‐1 and BGC823 cells (Figure 5A,B). After AGS cells were transfected with FoxO4 lentivirus, CDX2 protein and mRNA levels decreased as the relative exogenous p‐FoxO4 level increased (Figure 5C). These results illustrate that CDX2 expression was negatively regulated by p‐FoxO4.

### 3.6 Resveratrol is able to activate FoxO4 through the PI3K/AKT pathway

**FIGURE 6:**
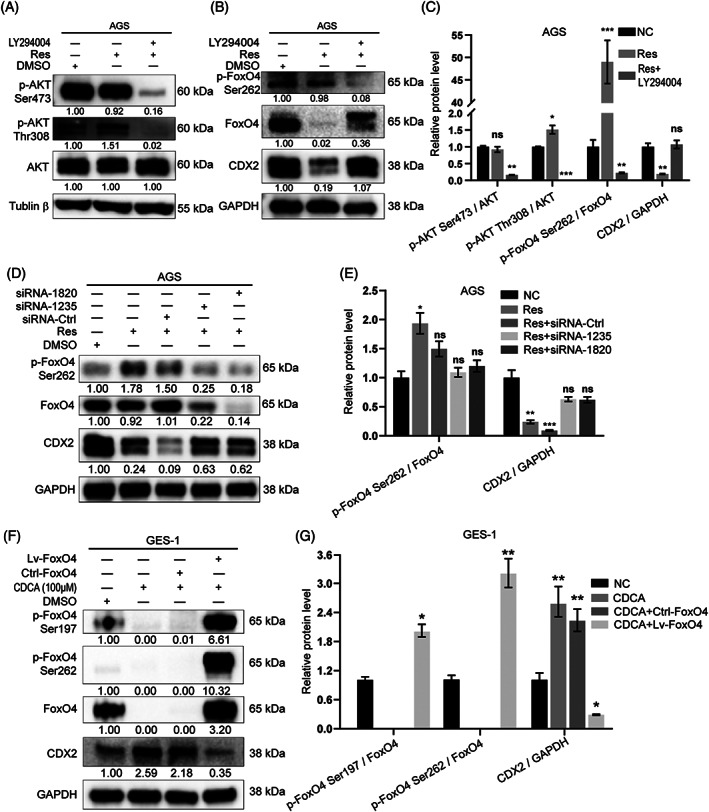
Resveratrol activated FoxO4 through the PI3K/AKT pathway. (A,B) The PI3K/AKT pathway inhibitor LY294004 partially blocked the effect of resveratrol on CDX2 at the protein level. (C) Relative protein levels of p‐AKT Ser473, p‐AKT Thr308, p‐FoxO4 Ser262 and CDX2 after treatment with the inhibitor LY294004. (D) AGS cells were transfected with siFoxO4. After a relative decrease in p‐FoxO4 resulted from the absolute reduction in FoxO4, the ability of resveratrol to reduce CDX2 weakened. (E) Relative protein levels of p‐FoxO4 Ser262 and CDX2 after siFoxO4 transfection. (F) GES‐1 cells were transfected with FoxO4 lentivirus. The relative increase in p‐FoxO4 suppressed the up‐regulation of CDX2 induced by CDCA. (G) Relative protein levels of p‐FoxO4 Ser262 and S197 and CDX2 after Lv‐FoxO4 transfection

To investigate the mechanism of FoxO4 activation after resveratrol treatment, the inhibitor PI3K/AKT pathway LY294004, siFoxO4 and Lv‐FoxO4 were used. LY294004 blocked the phosphorylation of AKT at Ser473 and Thr308 to block the PI3K/AKT pathway (Figure 6A). It further reduced the phosphorylation of FoxO4, thereby reducing the decreasing of CDX2 by resveratrol (Figure 6B). The relative expression changes in p‐AKT Ser473, p‐AKT Thr308, p‐FoxO4 Ser262 and CDX2 clearly indicated the relationship between p‐AKT and p‐FoxO4 (Figure 6C). To explore the effect of resveratrol on CDX2 in the absence of p‐FoxO4, siFoxO4 was transfected into AGS cells. Resveratrol weakened the reduction in CDX2 levels in the absence of p‐FoxO4 (Figure 6D). The relative expression changes in p‐FoxO4 Ser262 and CDX2 clearly indicated the relationship between p‐FoxO4 and CDX2 (Figure 6E). GES‐1 cells were transfected with Lv‐FoxO4 to observe the relationship between CDCA and p‐FoxO4. The absolute increase in p‐FoxO4 weakened the effect of CDCA on CDX2 (Figure 6F,G). These results revealed that resveratrol can activate FoxO4 through PI3K/AKT pathway activation.

### 3.7 p‐FoxO4 and CDX2 showed a reverse correlation in normal and GIM tissue array

**FIGURE 7:**
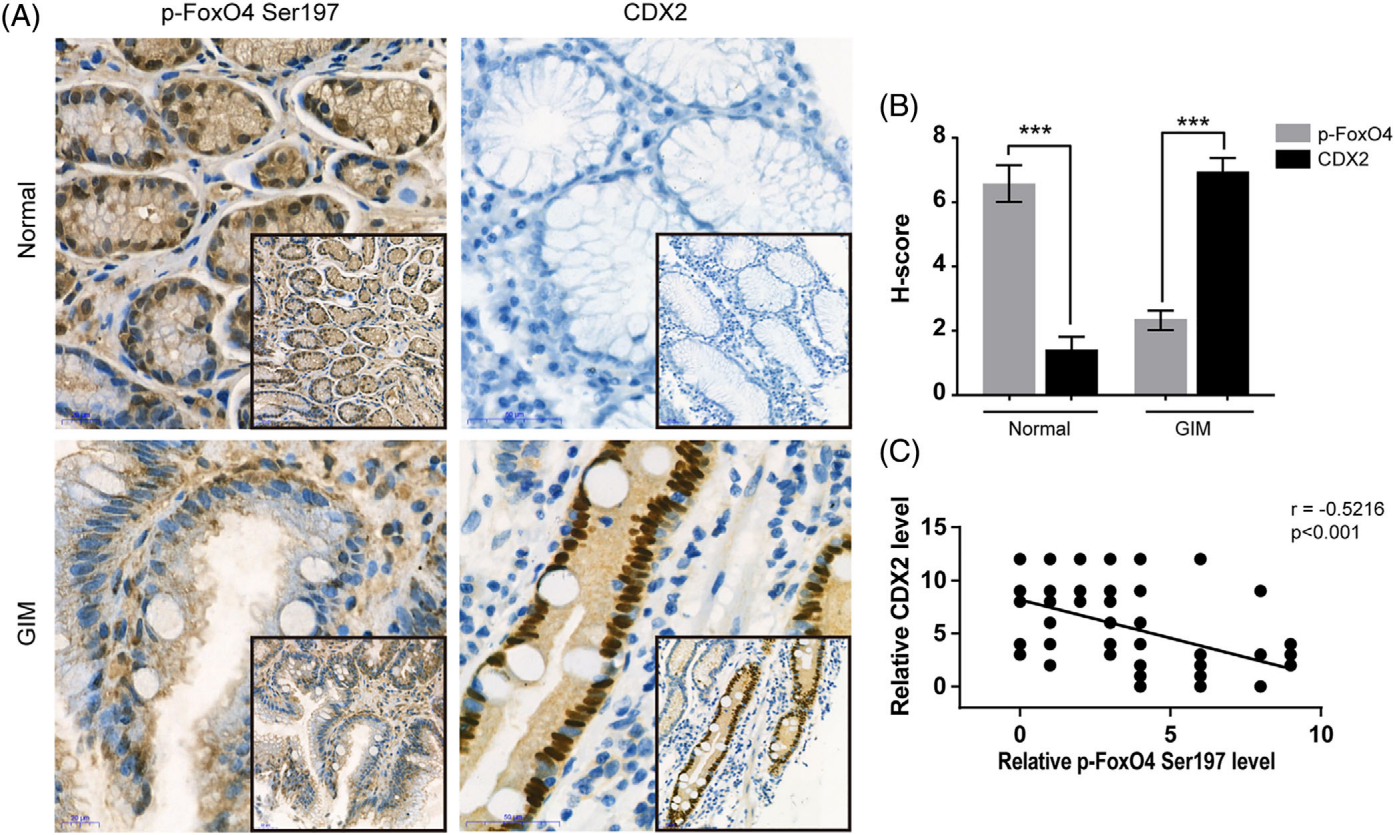
The PI3K/AKT/p‐FoxO4/CDX2 pathway is characteristic in normal and GIM tissue arrays. (A) Representative images of immunohistochemistry (IHC) staining for p‐FoxO4 Ser197 and CDX2 in normal tissues (upper) and IM tissues (lower) in 12 normal and 49 IM unpaired tissues. Scale bars: 20 μm. (Picture zoom 400 X; Embedded graphics 200X) (B) Relative protein levels of p‐FoxO4 S197 and CDX2 in normal (6.583 ± 0.570, n = 12; 1.417 ± 0.398, *n* = 12; Wilcoxon matched pairs, *p* value = .0005) and IM (2.367 ± 0.301, *n* = 49; 6.959 ± 0.464, *n* = 49; paired *t* test, *p* value <.0001) tissues detected by IHC. (C) Negative correlation between p‐FoxO4 Ser197 and CDX2 levels in normal and IM tissues (*n* = 61, *r* = −.5216, *p* value <.0001) [Colour figure can be viewed at wileyonlinelibrary.com]

Finally, to examine whether the above‐described regulation in gastric cell lines is clinically relevant, IHC for p‐FoxO4 and CDX2 was applied to 12 normal tissues and 49 IM tissues. Compared with that in normal tissues, CDX2 was significantly increased in IM tissues. The p‐FoxO4 levels were decreased in IM tissues compared with normal tissues (Figure 7A,B; normal: 6.583 ± 0.570, *n* = 12; 1.417 ± 0.398, *n* = 12; Wilcoxon matched pairs, *p* value = .0005; IM: 2.367 ± 0.301, *n* = 49; 6.959 ± 0.464, *n* = 49; paired *t* test, *p* value <.0001). In addition, the expression of CDX2 and p‐FoxO4 showed a reverse correlation (Figure 7C; *n* = 61, *r* = −.5216, *p* value <.0001). In summary, these results showed that the PI3K/AKT/p‐FoxO4/CDX2 pathway is inactive in human gastric IM.

## 4 DISCUSSION

In this study, we discovered a resveratrol‐induced pathway involving active AKT and downstream FoxO4 activated by phosphorylation. The pathway antagonized the induction of CDX2 by bile acids and may contribute to the treatment of IM.

Currently, the function of CDX2 in regulating intestinal differentiation is widely accepted. Furthermore, the previous work of our group has focused on the molecular mechanisms driving CDX2 and its downstream intestine‐specific marker expression by bile acids in the oesophagus and stomach, which include the FXR/SHP/NF‐κB pathway (Zhou et al., 2018), the miR‐92a/FOXD1/NF‐κB pathway (Li et al. 2019) and the HNF4α/HDCA6/CDX2 pathway (under review). Our previous study provides evidence that fractions of bile acids induce an intestine‐like phenotype in gastric cell lines. CDCA can well induce the increase of intestinal markers at 100 μM concentration (Li et al. 2019). Here, our study confirmed that resveratrol could prevent the increase in CDX2 induced by bile acids. GES‐1 cells were used to mimic gastric epithelial cells suffering from bile acids reflux (Li et al. 2019). Nevertheless, in the near future, a more persuasive model using primary human gastric cells will be required. Together with our previous study, our results show that bile acids can promote increases in CDX2 and intestinal markers.

Through review and analysis, resveratrol, a polyphenolic compound contained in red wine, was shown to have antioxidant, anti‐inflammatory and anti‐tumour effects (Chassot et al., 2018; Huminiecki & Horbańczuk, 2018; J. Xu et al., 2017; Y. Zhang et al., 2019). Most studies have shown that the target of resveratrol is generally Sirt1 (T. Liu et al., 2017; Wood et al., 2004). As a pharmacological agent, resveratrol has a wide spectrum of targets, whose biological activities may thus be dependent on its simultaneous activity on multiple molecular targets (Pirola & Frojdo, 2008). Here, active FoxO4, a common target of resveratrol and bile acids, has been the focus of our research. The Forkhead box O (FoxO) transcription factor family contains four related members: FoxO1, FoxO3, FoxO4 and FoxO6 (Daitoku, Sakamaki, & Fukamizu, 2011; Maiese, Chong, & Shang, 2008; Sykes et al., 2011). We previously discovered the role of Fox4 as a negative regulator of GC and CRC (X. Liu et al., 2011; Su et al., 2014). Phosphorylation by PKB/AKT1 inhibits transcriptional activity and is responsible for nuclear localization and accumulation (Mandai et al., 2018; F. Zhang, Virshup, & Cheong, 2018). In our study, we found that resveratrol could increase p‐FoxO4 and nuclear accumulation, which inhibited CDX2 transcription by binding directly to the promoter of CDX2. Cellular immunofluorescence confirmed this result. However, other studies identified that activation of the PI3K/AKT or MAPK pathway induced FoxO4 phosphorylation and resulted in its export from the nucleus into the cytoplasm with a reduction in DNA‐binding activity (Roy, Srivastava, & Shankar, 2010; Takaishi et al., 1999). These findings provide evidence that p‐FoxO4 may play an essential role in promoting the progression of IM due to bile acid reflux.

Notably, PI3K/AKT and MAPK are oncogenic signalling pathways that are usually activated in various cancers and target FoxO4 in similar ways to inhibit its anti‐cancer function. Similarly, isoorientin and momordin Ic induced cell death by up‐regulating FoxO4, mediated by inhibition of the PI3K/AKT and MAPK pathways in human hepatoblastoma cancer cells (Wang et al., 2014; Yuan et al., 2012). In our study, inhibition of the PI3K/AKT cascade by LY294004 decreased FoxO4 phosphorylation and nuclear accumulation, and subsequently increased CDX2 expression. When p‐FoxO4 was reduced by 75% or less, the inhibitory effect on CDX2 could be ablated. Conversely, if the absolute content of p‐FoxO4 is increased, CDX2 transcription will be inhibited. However, other experimental studies suggest that inhibiting the PI3K/AKT cascade by LY294002 increased FoxO4 transcription, which might be caused by impaired FoxO3 phosphorylation and thus results in increased FoxO3 activity (Franz et al., 2016). Furthermore, acute starvation or caloric restriction has been shown to trigger an increase in FoxO4 mRNA levels in skeletal muscles (Furuyama et al., 2002; Mofarrahi et al., 2014). Together with our results, these findings provide evidence that FoxO4 may be activated by resveratrol via PI3K/AKT activation.

In summary, we propose a schematic model of gastric IM development (Figure S2). This figure illustrates that in gastric epithelial cells, bile acid‐stimulated IM induces the up‐regulation of CDX2 and downstream intestine‐specific markers via the miR‐92a‐1‐5p/FoxD1/NF‐kB pathway, the FXR/SHP/NF‐kB pathway and FoxO4/CDX2 pathway. Resveratrol induces up‐regulation in p‐AKT, leading to an increase of p‐FoxO4 and nuclear accumulation, which inhibits CDX2 transcription and IM formation. This new PI3K/AKT/p‐FoxO4/CDX2 pathway may help clarify the mechanism underlying the regulatory effects of resveratrol in gastric IM and shed new light on the early prevention and reversal of gastric IM.

## CONFLICT OF INTEREST

The authors declare no conflicts of interest.

## Supporting information


**Figure S1.** The quantitative analysis data of fluorescence intensity. (a) The relative fluorescence intensity of CDX2 in Figure 1c; (b) The relative fluorescence intensity of FoxO4/p‐FoxO4 in Figure 3e,f; (b) The relative fluorescence intensity of FoxO4/p‐FoxO4 in Figure 3g,h. *p < .05; **p < .01; ***p < .001; * Compared with Control group; #*p* < .05; ##*p* < .01; ###*p* < .001; # Compared with CDCA group; ns, not significant.

**Figure S2.** A schematic model of the PI3K/AKT/p‐FoxO4/CDX2 pathway in gastric cells. In response to bile acid reflux, the transcription of CDX2 and downstream intestinal markers is upregulated through the miR‐92a/FOXD1/CDX2 and FXR/SHP/NF‐kB pathways. In contrast, after treatment with resveratrol, PI3K/AKT activation promotes FoxO4 phosphorylation, which increases its nuclear translocation and suppresses CDX2 transcription, resulting in the downregulation of intestinal markers including Villin1, cadherin17, and Kruppel‐like factor 4 (Klf4).

**Table S1.** Overlapping targets of the transignal protein/DNA array kit.
**Table S2.** Overlapping targets of the Cignal Finder 45‐pathway arrays.

**Table S3.** The relative change folds post‐treatment of CDCA.
**Table S4.** The relative change folds post‐treatment of resveratrol.
**Table S5.** Bioinformatics prediction combined with CDX2 promoter region.
