# Supplementary figures and images for: Resveratrol inhibits bile acid‐induced gastric intestinal metaplasia via the PI3K/AKT/p‐FoxO4 signalling pathway
Source: Phytother Res. 2020 Oct 25;35(3):1495–507. doi: 10.1002/ptr.6915 (PMC8048559; doi:10.1002/ptr.6915)

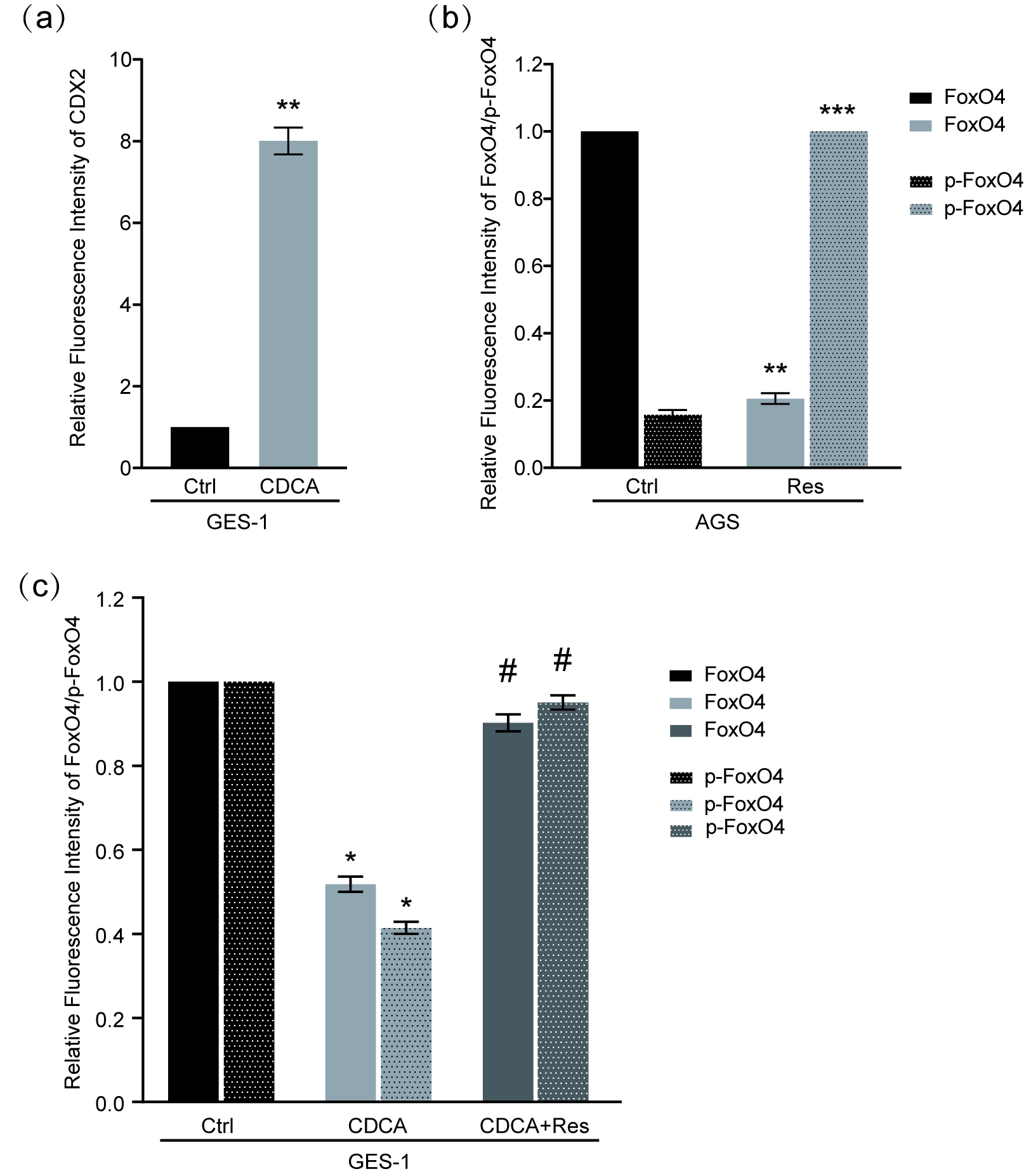

Supplement: Supplementary file 1 — Figure S1. The quantitative analysis data of fluorescence intensity. (a) The relative fluorescence intensity of CDX2 in Figure 1c; (b) The relative fluorescence intensity of FoxO4/p‐FoxO4 in Figure 3e,f; (b) The relative fluorescence intensity of FoxO4/p‐FoxO4 in Figure 3g,h. *p < .05; **p < .01; ***p < .001; * Compared with Control group; #p < .05; ##p < .01; ###p < .001; # Compared with CDCA group; ns, not significant. [file PTR-35-1495-s001.tif]

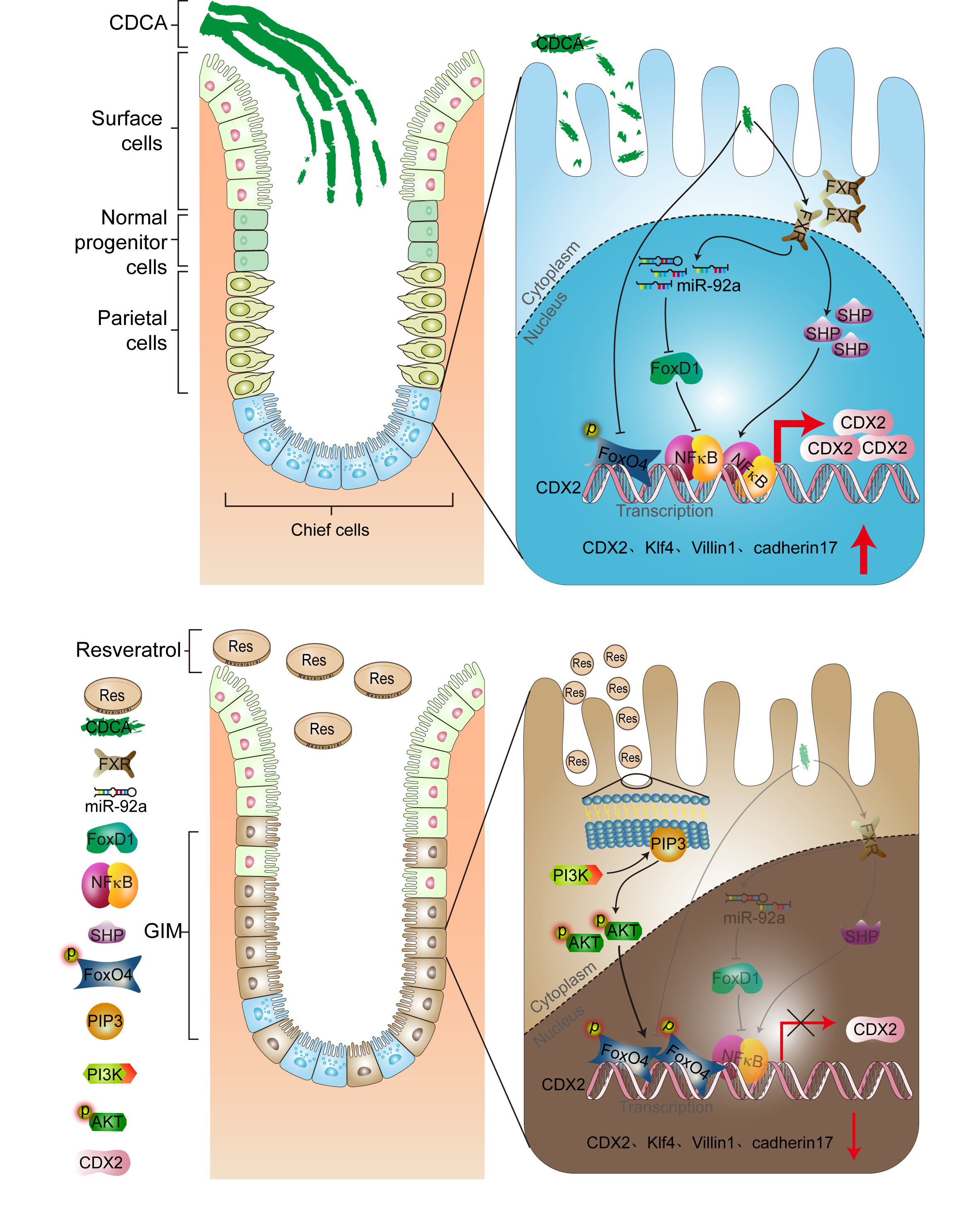

Supplement: Supplementary file 2 — Figure S2. A schematic model of the PI3K/AKT/p‐FoxO4/CDX2 pathway in gastric cells. In response to bile acid reflux, the transcription of CDX2 and downstream intestinal markers is upregulated through the miR‐92a/FOXD1/CDX2 and FXR/SHP/NF‐kB pathways. In contrast, after treatment with resveratrol, PI3K/AKT activation promotes FoxO4 phosphorylation, which increases its nuclear translocation and suppresses CDX2 transcription, resulting in the downregulation of intestinal markers including Villin1, cadherin17, and Kruppel‐like factor 4 (Klf4). [file PTR-35-1495-s004.tif]
