# Supplementary material for: Resveratrol inhibits bile acid‐induced gastric intestinal metaplasia via the PI3K/AKT/p‐FoxO4 signalling pathway
Source: Phytother Res. 2020 Oct 25;35(3):1495–507. doi: 10.1002/ptr.6915 (PMC8048559; doi:10.1002/ptr.6915)
Supplement: Supplementary file 3 — Table S1. Overlapping targets of the transignal protein/DNA array kit. Table S2. Overlapping targets of the Cignal Finder 45‐pathway arrays. [file PTR-35-1495-s002.docx]

**Supplementary material_1.** Overlapping targets of the TranSignal Protein/DNA Array Kit

| AP-1 | Brn-3 | CDP | E2F-1 | Ets |
| --- | --- | --- | --- | --- |
| GAS/ISRE | IRF-1 | Myc/Max | NF-E1 (YY1) | oct-1 |
| Pbx1 | PRE | SIE | Sp1 | Stat3 |
| Stat5/Stat6 | TR(DR-4) | HSE | AhR/Arnt | AP3 |
| CdxA/NKX2 | CETP-CRE | c-Rel | E4F, ATF | FKHR |
| Freac-2 (1) | Freac-7 | GATA-1 | GATA-3 | Gfi-1 |
| HFH-3 | HNF-3 (a, b, g) | HNF-4 & COUP-TF | ISRE (1) | KLF |
| hTERT-MT-Box | NF-Y | NZF-3 | PARP | Pax4 |
| PPARa | RSRFC4 | SRY | XBP-1 | AF1, ARP1., NF-BA |
| AML1 | ATF/CRE | CCAAT | CD28RC, NF-IL2B | CP1, CTF, CBTF |
| CSBP | EBP40, 45 | Elf-1 | HiNF | HNF-1A |
| HOXD8 | ICSBP | KTP1 | LR1 | LyF-1 |
| MTF | MyTI | NF-4FA | NF-Y | p55 |
| PPUR(1) | PYR | RIPE3a1 | SIF3 | TCE |
| TGT3 | v-Maf | WT1 (3) | ZNF174 | ACF |
| alpha-PAL | ATF-adelta | BZP | C/EBPalpha(2) | CBF |
| c-myb binding protein | CP1B | CYP1A1 | E2 | EGR1 |
| ETF | GBF1/2/3/HY5 | HFH-11B/11a | HiNF-B/H1TF1 | HNF-4alpha2/1 |
| ISGF | LF-A1(2) | LH2/Lim1 | MBP-1(1) | MDBP(2) |
| MHC gene promoter W box | myc-CF1 | NF-1(2) | NFAT-1 | NF-E2(2) |
| NFkBp65 | ODC | pax2 | PBGD binding protein | PO-B |
| PREB | PU.1 | RORE | SPERM1 | Stat1/Stat3 |
| T3R | TEF1(2) | Thy-1binding protein | TxREF, NF III | WAP BP |
| AP-2 | C/EBP(a, b & other) | c-Myb | EGR | Ets-1/PEA3 |
| GATA | MEF-1 | NF-1 | NF-E2 | p53 |
| Pit 1 | RAR(DR-5) | Smad SBE | SRE | Stat4 |
| TFIID | USF-1 | MRE | Angiotensinogen ANG-IRE | AP4 |
| CEF1 | COUP-TF | E47 | Elk1 | Fkhr |
| Freac-2 (2) | GAG | GATA-1/2 | GATA-4 | HFH-1 |
| HFH-8 | HNF-3 b | Ikaros | ISRE (TRANSFAC) | MEF-3 |
| MUSF1 | Nkx-2.5 | ORE | Pax2 | Pax6 |
| PPARr | SAA | Tax/CREB | XRE | AFP1 |
| AREB6 | ATF2 | CCAC | Cdx2 | CPE |
| CTCF | EKLF(1) | HEN1 | HLF | HOX4C |
| HOXD8 | Isl-1 | LCR-F1 | LyF | MAZ |
| MyoD | MZF1(1) | NF-Gma (1) | NRF-1 | PEBP |
| PPUR(2) | RB | SIF1 | SP1, ASP | TFE3 |
| TTF1 | WT1 (1) | X2BP | AAF | ACPBP(CRY-alphaA) |
| AP2 | ATF-a | C/EBPa/g | C/EBPgamma | c-Ets-1 |
| c-Myc-responsive region | CP1 | DE I | EBP-80 | EGR2 BP |
| Fra-1/JUN | GKLF | HFH | HiNF-D3 | HOXA4 |
| kBF-A | LF-A2 | LSF | MBP-1(2) | MEF-2a |
| msx1/2/3 | myc-PRF | NF-1/L | NF-Atp | NFE-6/CP1 |
| N-ras binding protein | p53(2) | Pax5(2) | PCF | PRDI-BFc |
| PTF1-beta | PUR | RVF | SRF(2) | Stat5b |
| Tat | TFE3-L | transferrinBP | URE | XBP1(2) |
| ARE | CBF | CREB | ERE | FAST-1 |
| GRE | MEF-2 | NFATc | NFkB | Pax-5 |
| PPAR | RXR(DR-1) | Smad 3/4 | Stat1 (p84/p91) | Stat5 |
| TR | VDR(DR-3) | ADR1 | Antioxidant RE | Beta-response element |
| CEF2 | CREB-BP1 | E4BP4 | EVI-1 | Afxh (Foxo4) |
| Freac-4 | GAS | GATA-2 | GATA-6 | HFH-2 |
| HIF-1 | HNF-4 (2) | IRF-1, IRF-2 | L-III BP | MSP1 |
| MZF1 | NPAS2 | p300 | Pax3 | Pax8 |
| RREB (1) | Skn | TCF/LEF | ADD1 | AIC, CBF |
| ARP | CACC | CD28RC | CEA | CREB2 |
| E12 | EKLF(2) | HFH-8, HNF3,LUN | HMG | HOXD8,9,10 |
| HOXD9,10 | KPF1 | LF-A1 | LyF-1 | MTB-Zf |
| MyoG | MZF1(2) | NFIL-2 | p53 | PEBP2 |
| Pur-1 | RFX1,2,3, | SIF2 | SRF, SAP | Tf-LF |
| TREF1, 2 | WT1 (2) | XBP1, X2BP | ABF-1 | ALF1B |
| AP3 | beta M-globin factor B1 | C/EBPalpha(1) | CBFB | c-fos enhancer-binding protein |
| COUP-TF(2) | CREB(2) | E12/E47 | EGF binding protein | EIL1/2/3 |
| GATA1(2) | H4TF-1 | HiNF-A | HNF1a/b/c | IL-6 RE-BP |
| lactoferrin BP | LF-B2 | LXRE1 | MDBP(1) | Mfh-1 |
| Myb(2) | NCAM binding protein | NF-A3 | NF-Atx | NFkB(2) |
| Oct-4/Oct3 | PAX1 | Pax-6 | PEPCK promoter GR | PRDII-BF1 |
| PTF1 | RFX1/2/3 | Snail | SSAP | Surf-2(2) |
| TEF1(1) | TFEB | TTF-1(1) | v-rel 50-55K | YB1 |

**Supplementary material_2.** Overlapping targets of the Cignal Finder 45-Pathway Arrays

| ATF2/3/4 | ER | STAT1 | Oct4 | STAT3 |
| --- | --- | --- | --- | --- |
| AR | GATA | KLF4 | Pax6 | SMAD2/3/4 |
| Nrf2/Nrf1 | GR | LXR | FOXO | VDR |
| ATF6 | HSF-1 | SRF/EIk-1 | NFAT | TCF/LEF |
| C/EBP | MTF-1 | AP-1 | PPAR | AhR |
| CREB | Gli | MEF2 | PR |  |
| E2F | HNF4 | c-Myc | RAR |  |
| P53 | HIF-1a | Nanog | RXR |  |
| EGR1 | IRF1 | RBP-Jk | Sox2 |  |
| CBF/NF-Y/YY1 | STAT1/STAT2 | NFkB | SP1 |  |
